# Quality of life and physical activity in type 1 diabetes

**DOI:** 10.1186/s12887-025-05632-6

**Published:** 2025-05-01

**Authors:** Emma J. Cockcroft, Dillon Chithuranjan, Parth Narendran, Robert C. Andrews, Renuka P. Dias

**Affiliations:** 1 University of Exeter Medical School, Exeter, EX1 2JP UK; 2 Institute of Applied Health Research, University of Birmingham, Birmingham, UK; 3 Institute of Immunology and Immunotherapy, University of Birmingham, Birmingham, UK; 4 Department of Diabetes, University Hospitals Birmingham NHS Foundation Trust, Birmingham, UK; 5 Department of Diabetes, Taunton and Somerset NHS Foundation Trust, Taunton, UK; 6 Department of Paediatric Endocrinology and Diabetes, Birmingham Women’s and Children’s NHS Foundation Trust, Birmingham, UK; 7 Institute of Applied Health Research, College of Medical and Dental Sciences, University of Birmingham, Birmingham, UK

**Keywords:** Type 1 diabetes, Physical activity, Quality of life

## Abstract

**Introduction:**

T1 Diabetes (T1D) is one of the most common chronic diseases in children and young people [1] with almost 34 000 aged 18 years or less living with T1D [2]. Physical activity is promoted as one of a number of management tools for people living with diabetes, being associated with significant health benefits including improved glycaemic control. The benefits of physical activity on quality of life in children with T1D is unclear with confounding effects of disease duration and co-morbidities in studies.

**Aim:**

To determine the effect of physical activity interventions on quality of life of children with type 1 diabetes.

**Methods:**

A systematic review was conducted and reported in line with PRISMA 2009 guidance. The CINAHL, Embase, International Pharmaceutical Abstracts, Medline, PubMed and PsychINFO databases were searched for the period January 1994 to March 2025. Papers were included in the present review if they included a study intervention in children under 19 years of age that was more than a single exercise session and had a control group (with or without T1D) as a comparator group. The primary outcome measure was Quality of Life (QoL) indicators.

**Results:**

We assessed 3020 records, of which three randomised controlled trials (RCT) published between 2007 and 2020 met study inclusion criteria. There was significant heterogeneity in study design, methods and reporting. Benefits of physical activity were not consistently seen across studies.

**Conclusion:**

There remains limited data on QoL outcomes or even a standardisation for measuring QoL in this cohort as seen by the various validated tools used across studies. There continues to be a need for further work to understand the additional framework (psychological underpinning) to cause longer term impactful changes on both physical and psychological health in children with T1D.

**Supplementary Information:**

The online version contains supplementary material available at 10.1186/s12887-025-05632-6.

## Introduction

Type 1 Diabetes (T1D) is one of the most common chronic diseases in children [1]. Currently there are almost 30,000 children (under 18 years of age) living with T1D in the UK [3]. The complex treatment regimen, despite advances in automated insulin delivery devices, also known as hybrid closed loop systems, requires up to several hundred additional decisions per day for an individual living with T1D or their carer, and is thought to have impacts of long-term quality of life (QoL) [4, 5].

QoL is a multidimensional concept consisting of subjectively indicated wellbeing in a range of domains. QoL is defined by the World Health Organization (WHO) as “an individual’s perceptions of their position in life, in the context of the culture and value systems in which they live, and in relation to their goals, expectations, standards, and concerns” [5]. QoL is impacted by the diagnosis of T1D in children with a number of studies reporting worse health-related QoL scores across a number of domains [6, 7]. Children living with face not only the universal transitional challenges across developmental milestones but are also expected to take many decisions each day related to their diabetes care. They are expected to have a high degree of self-management and an awareness of suboptimal glycaemic control increasing the risk of disease complications [8].

It is well recognized that people living with T1D are pre-disposed to several micro-vascular and macro-vascular co-morbidities, linked to duration of diabetes and long-term glycaemic control. People with T1D have a significantly higher risk of cardiovascular disease compared to those who do not have diabetes [9].

Physical activity in children is associated with several benefits, both physical and psychological [10, 11]. In individuals with T1D additional benefits are seen, including increased insulin sensitivity and improvements in glycaemic control, lipid profile, and body composition [12–14]. Physical activity is also associated with a reduction in the long-term cardiovascular risk linked to T1D [12]. In children without diabetes increased physical activity is associated with improvement in wellbeing measures across a number of domains [13, 14].

Due to the associated benefits, a number of organisations including the WHO and International Society for Pediatric and Adolescent Diabetes (ISPAD) recommend that children with T1D should perform physical activity for a minimum of 60 min per day, including vigorous physical activity for a minimum of 20 min, and they should minimise sedentary time each day [15]. Despite these guidelines, studies suggest that the majority of children with T1D are not meeting recommended levels of physical activity and are significantly less physically active compared to peers without T1DM [16, 17].

Due to evidence of lower physical activity levels in children with T1D compared to healthy peers [18], and the potential health benefits, there have been several studies assessing the effectiveness of physical activity interventions on health related outcomes in children with T1D [12, 19, 20]. These have all shown improvements in glycaemic control with physical activity interventions as well as several secondary physical health outcomes.

In children without T1DM, physical activity interventions have been shown to improve overall QoL [13]. However the relationship between physical activity and QoL in children with T1D is less clear [21] with the potential to increase management burden and negatively affect QoL [22]. Despite evidence from several systematic reviews providing health-related data, none have focused on how interventions may affect overall QoL for children living with T1D [12, 19]. It is important to understand the impact of physical activity interventions on QoL to ensure that interventions consider overall wellbeing and not just cardiometabolic outcomes, offering a more patient-centred and holistic approach.

This systematic review aims to synthesis the evidence from randomised controlled trials (RCTs) that have assessed effectiveness of physical activity interventions on quality of life of children with type 1 diabetes. Additionally, the review aims to review the reporting of QoL outcomes in RCTs and provide recommendation for future research around intervention effectiveness assessment.

## Methods

A systematic review of RCTs was conducted. The protocol registered on the International Prospective Register for Systematic Reviews (PROSPERO) (Registration number: CRD42023440626). The conduct and reporting of the current systematic review is in accordance with the Preferred Reporting Items of Systematic Reviews and Meta-Analysis (PRISMA) guidelines [23].

### Search strategy

Six databases (MEDLINE, EMBASE, PsycINFO (via Ovid), CINAHL (Via EBSCO) and Cochrane Library) were systematically searched for relevant citations published from [1946-March 15 2025]. The search strategies were developed by members of the research team with assistance from an information specialist and informed by refining previous systematic reviews in the area [20, 24]. Free-text and medical subject heading (MeSH) terms were combined using Boolean operators “OR” and “AND” to develop a comprehensive search strategy relating to population and intervention of interest (see Fig. S1).

### Inclusion and exclusion criteria

Eligible studies were determined though the Patient, Intervention Comparator, Outcome and Study Design (PICOS) framework with the following concepts:

#### Population

Children clinically diagnosed with T1D, up to age 19 years [2]. If studies included young people both within and outside our specified age range, we only included them in the analysis if we were able to separate data based on age groups.

#### Intervention

Physical activity in the form of an exercise training program, which had to consist of more than a one-off exercise session. Physical activity interventions could be delivered in any modality, by any individual(s), and at any location.

#### Comparator

A comparison group of children with T1DM who were not advised to do any additional exercise (either supervised training sessions or unsupervised sessions).

#### Outcome

Quality of life (QoL) measured by any validated form.

#### Study design

Randomised controlled trials (RCT).

Additionally, studies had to be peer reviewed articles published in English.

### Study selection

Retrieved citations were uploaded into review management system Covidence (Veritas Health Innovation), and duplicates were removed. Two independent reviewers (RPD and EC) completed screening for both titles, abstracts and full-text articles based on the prespecified PICOS criteria. Disagreement between authors was resolved independently by a third author.

### Data extraction and management

One author (DC) extracted data from the included manuscripts using a data extraction form developed for this review. A second author (EC and RPD) verified data accuracy. Data were collected on study design, participant characteristics (age, gender, baseline glycaemic control), intervention and control group description (type of physical activity, frequency, duration, supervision), and outcomes measures.

### Risk of Bias assessment

**Fig. 1:**
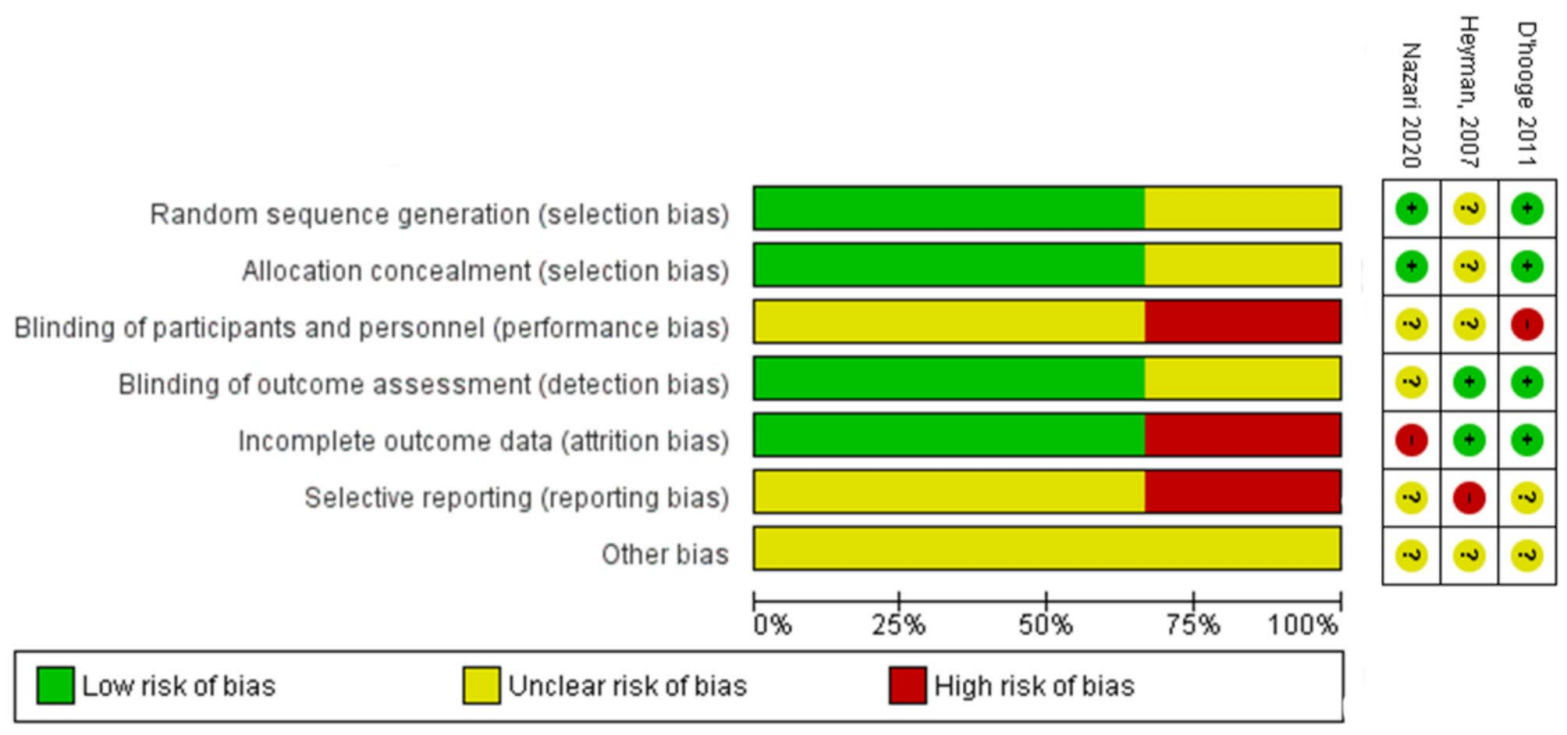
Risk of bias on included studies

Two reviewers (EC and DC) independently assessed the quality of individual trials using six domains of the Cochrane Collaboration’s tool for assessing risk of bias (Fig. 1) [25]including sequence generation; allocation concealment; blinding of outcome assessors; completeness of outcome data; selective reporting of outcomes; and other sources of bias. For each domain, studies were classified as being at low, high or unclear risk of bias. Disparities during the checking process were resolved through discussion.

### Data synthesis and analysis

Due to the limited data and variability in outcomes assessment measure we were unable to conduct a meta-analysis. We therefore summarised results using descriptive statistics and narrative synthesis in line with the ‘synthesis without meta-analysis’ (SWiM) guidelines [26] to report the use of QoL outcomes and the effect of PA interventions on QoL.

## Results

**Fig. 2:**
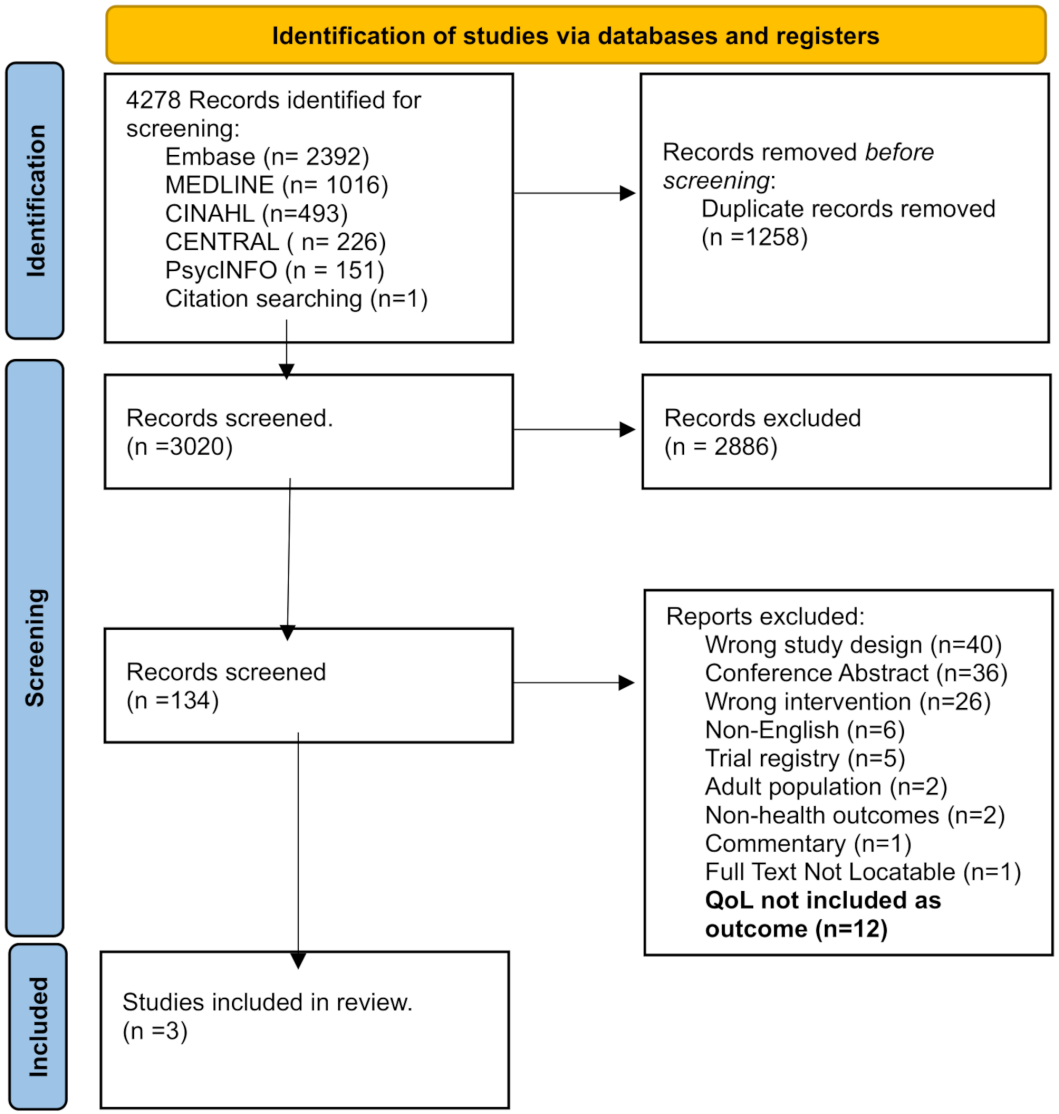
PRISMA flow diagram

The search of the relevant databases identified a total of 4278 studies for screening, with 3020 left after duplicates had been removed (Fig. 2). In total 134 studies were screened at the full text level, with 131 being excluded (reasons for exclusion reported in Fig. 2). Two of the 131 excluded studies were excluded as we were not able to extract only the paediatric data from the studies which included a mixed of adults and children. A total of 15 studies were RCTs of physical activity interventions assessing health outcomes, of which only 3 measured QoL.

The studies were conducted between 2007 and 2020 in France [27], Iran [28]and Belgium [29].

### Participant characteristics

A total of 72 participants below 19 years of age (26 males and 46 females) were included in this review. Two studies included both males and females [28, 29], and 1 study only included female participants [27]. The mean age of was 12.7 years (SD 2.8) across intervention and control groups.

All patients were recruited in hospitals or in diabetes clinics. Every study was composed of an intervention group and a control group.

At baseline, the mean HbA1c levels of the patients were 7.9% +/- 1.3% 62.8mmol/mol (+/-14.2 mmol/mol). The mean diabetes duration was 6.4 years +/- 3.3 SD (given for 2 papers) [27, 29].

### Interventions

All three studies combined aerobic and anaerobic physical activities. All the studies aimed for moderate-vigorous intensity physical activity. In all studies, the charge of exercises increased along the studies. Physical fitness scores improved significantly in all the intervention groups compared to baseline and controls which alongside attendance data (see Table 1) is indicative that training was adhered to. All training sessions were supervised by educators or physiotherapists in 2 studies [28, 29] whilst one study split sessions into one supervised and one unsupervised each week [27].

**Table 1:** Summary of included studies

Author (Year)	No. of participants	Setting (Country)	Intervention (length in weeks)	Programme Intensity (per week) and adherence	Comparison Group	Age/Gender	Outcome Measures
D’hooge et al., 2011	16	Hospital (Belgium)	Supervised Aerobic and Weight Training (20 weeks)	2 × 70 min Aerobic − 60–75% MHR Resistance − 20RM-12RM **Adherence**: 38 training sessions were organized. The median number of session participations was 24 with a minimum of 20 and a maximum of 32	No training given (T1D) Recruitment: Patients and controls from Paediatric Diabetes clinic (University of Ghent)	10–18 (Males and Females)	BMI HbA1c VO2 max 6 min walk test Strength measures **QoL (SF-36)**
Nazari et al., 2020	40	Community (Iran)	Supervised Resistance and Aerobic Training Program (16 weeks)	3 × 60 min Aerobic − 50–75% MHR for 10–20 min Resistance − 2–3 sets of 8–12 reps **Adherence**: Not reported but does state that subjects would be “excluded from the study if they were absent for more than three sessions”	No training given (T1D) Recruitment: Patients and controls recruited 17-Shahrivar Pediatric Hospital in Rasht	8–14 (Males and Females)	BMI **QoL (RCMAS and PedsQol)**
Heyman et al., 2007	16	(1) Home (unsupervised sessions) (2) Training Facility (supervised sessions) (France)	Strength exercises and Aerobic Training (24 weeks)	**2 × 60 min** (1) Supervised session: Combination of aerobic (intermittent workloads) and strength exercises (ratio 2/1) workload 80 to 90% HRR (2) Unsupervised session: Specific recommendations for technique, duration, frequency and ways to avoid common errors workload 80 to 90% HRR **Adherence**: **100%**	No training given (T1D) Recruitment Patients and controls from Paediatric Endocrinology Unit (Rennes)	Post-menarche (< 18.5 years) Females	BMI Waist hip ratio VO2 max Lipid profile **QoL (DQoL)**

MHR- Maximal heart rate, RM- Repetition max, HRR – heart rate reserve, BMI – Body mass index, QoL – Quality of life, HbA1c - glycosylated hemoglobin, RCMAS- Revised Children’s Manifest Anxiety Scale, Pediatric Quality of Life (PedsQoL), DQoL – Diabetes related quality of life

In Heyman et al., aerobic exercises were varied throughout the 24 week period and included running, dance, step, football, volleyball, rock climbing and gymnastics. Additional strengthening exercises were included alongside aerobic activities. Activities were twice a week (including one session per week which was unsupervised) [27].

In Nazari et al., participants undertook interval training with resistance and weight-bearing exercises with Pilates followed by aerobic activities such as marching. The training programme occurred 3 times per week over 16 weeks [28].

In D’Hooge et al., young people undertook a supervised training session twice a week including aerobic (cycling, running, step) and strengthening exercises over 20 weeks [29].

### Risk of Bias within studies

Of the three included studies all were rated as “high” risk of bias. The risk of bias assessment in shown in Fig. 2. One study did not report details of the randomisation process. Two did not report details of blinding of outcomes assessment, and 1 reported limited blinding. One study had high risk of bias in selective reporting due to only reporting on subscale of QoL. One study did not provide reason for participant exclusion.

### Quality of life

#### Assessment of quality of life

All three included studies used different measure to assess quality of life. Only one used a QoL measure validated in children of all ages [28], and only one study used a QoL meaure using specific diabetes QoL outcomes [27].

D’Hooge and colleagues [29] used the Dutch version of general health survey short form (SF-36) [30, 31]. SF-36 is a 36 item self-report which measures perceived health in the areas of physical functioning, role-physical, bodily pain, general health, vitality, social functioning, role-emotional and mental health. SF-36 is designed as a general health measure tool but is often used for QoL assessment [31].

Nazari and colleagues [28] used The Paediatric Quality of Life (PedsQL) inventory to measure the children’s quality of life [32]. PedsQol is a 23 item questionnaire measured on Likert scale across 4 sections; physical functioning, emotional functioning, social functioning, and school functioning. PedsQoL has been shown to be both valid and reliable in the assessment of QoL in type 1 diabetes [33].

Heyman and colleagues [27] used a French translation of the Diabetes Quality of life questionnaire (DQOL) [34], which was modified for use with youth [35]. The questionnaire had 52 items across 4 sections: impact of diabetes, worries about diabetes, satisfaction with life (subscales: “with diabetes,” “in general”), and health perception.

#### Effect of intervention on quality of life

Of the three included studies two reported improvements to QoL in the intervention’s groups [27, 28].

Heyman and colleagues reported improved QoL only in the intervention group, as reflected by a clinically significant change (− 14.6 ± 55.5%) in the “satisfaction with diabetes” subscale score [36]. No between group differences and no detail of overall QoL assessment were reported.

Nazari and colleagues reported that the indices of quality of life and anxiety were decreased in the experimental group significantly (*p* = 0.001 and *p* = 0.003, respectively). They reported significant differences in these indices between the training and control groups (*p* = 0.003 and *p* = 0.001, respectively). Overall QoL post intervention was 80.08 (+/- 2.70) in exercise group and 75.93 (+/-) in control group (*p* = 0.003). This would be considered a clinically meaningful difference [37].

D’Hooge reported no significant effects on any of the different subdomains of the quality of life [29].

## Discussion

To our knowledge, this is the first systematic review to focus on the effects of physical activity interventions on QoL in children with T1D. This review builds on previous work with a general health outcome focus [20] and a more recent systematic review which did not include QoL outcomes [16, 19].

The main finding of this review was the limited quantity and poor quality of studies including QoL as an outcome’s measures. Additionally, findings suggest potential for physical activity interventions to improve domains of QoL, but due to inconsistent reporting firm conclusions cannot be made.

This review builds on previous reviews on the overall effects of physical activity interventions in children with T1D. Quirk and colleagues [20] reviewed evidence from both randomized and non-randomised quasi-experimental studies, and prospective cohort studies. They included 2 RCTS which included QoL outcomes ( [27, 29]both using different assessment methods. Similarly Absil and colleagues conducted a systematic review of RCTs focusing on the overall effects of physical activity [12]. Since these reviews were conducted only one additional RCT has been published with a relatively large sample size compared to previous studies (40 participants compared to 16). Given inconsistent assessment, poor quality studies and minimal observed effects we are unable to make a definitive conclusion on the effectiveness of physical activity interventions on QoL in this group and more research, using validated QoL assessment measures are needed.

Perhaps interesting is the finding that only a small number of studies included QoL as an outcome in physical activity intervention research, despite QoL being a key part of diabetes clinical care. Of the 15 RCTs of physical activity interventions in children with T1D only 3 included QoL. This is important as it has been suggested that interventions around physical activity in this group have the potential to decrease QoL due to additional treatment burden, which has been observed in other non-physical activity self-management interventions [38]. This absence of QoL measures seems to be ubiquitous across interventional research in both adults and children with T1D with only 4 out of 19 studies including QoL [39]. In the current review only one used diabetes specific measures of quality of life which is only validated in children 11 years and older. There is a lack of consistency in measurement and reporting of all QoL measures across studies as well as no inclusion measures specific to diabetes such as the PedsQoL diabetes module [33].

A recent review assessing the effects of physical activity interventions on QoL in children without chronic health conditions [13]concluded that physical activity interventions were an effective strategy from improving overall QoL in children. The observed effect was small (0.179 (0.045, 0.002)). Much like findings from the present review, authors noted inconsistent use of outcome measure with 5 measurement instruments used across 17 studies including KIDSCREEN, Paediatric Quality of Life Inventory, Child Health Questionnaire, and The Child Health Utility 9D questionnaires. The importance of QoL assessment is emphasised by groups such as the U.K Medical Research Council [40] and can play a key role in funding decisions via quality-adjusted life-year (QOLY) economic evaluation. If physical activity is to be ‘valued’ and funded then trials need to provide evidence of effect both in terms of physiological benefit as well as psychological and economical, which requires assessment of QOL.

### Strengths and limitations

This study has several limitations which should be considered. Firstly, due to limited data and heterogeneity of interventions and outcome assessment in included studies we were not able to perform a meta-analysis, which limited the overall assessment of the effects of physical activity interventions on QoL. Results have been reported narratively following reporting guidelines. Secondly, only articles published in English language were included and we therefore may have missed relevant studies in other languages. Thirdly, all the studies were deemed to be at high risk of bias and given the limited data available, firm conclusions regarding benefit or lack of thereof in QoL outcomes in children cannot be drawn.

## Conclusions

Our review suggests limited evidence for the meaningful improvement in QoL from physical activity interventions. We suggest this is in part due to limited assessment of QoL (measured only in 3 out of 15 published RCTS) as well as inconsistent assessment. There is no consensus on the most appropriate measurement tool for QoL in children with diabetes and the various questionnaires published add to the complexity in assessment [41]. Given the importance of QoL as an outcome we suggest that future research should increase focus on the effects of physical activity on QoL outcomes and coming to consensus regarding validated tools to assess QoL in children with diabetes. This can be addressed by studies including validated paediatric diabetes specific questionnaires, like the PEDsQoL, at various time points, enabling comparisons to be made at different stages of studies [33, 37], allowing meaningful assessment of the impact of interventions beyond traditional clinical markers.

## Electronic supplementary material

Below is the link to the electronic supplementary material.

Appendix S1: Deviations from the study protocol

Supplementary Figure 1: Search Strategy

## Data Availability

All data generated or analysed during this study are included in this published article.

## Declarations

### Datasets

All data generated or analysed during this study are included in this published article.
