# Supplementary material for: Quality of life and physical activity in type 1 diabetes
Source: BMC Pediatr. 2025 May 1;25:345. doi: 10.1186/s12887-025-05632-6 (PMC12044773; doi:10.1186/s12887-025-05632-6)
Supplement: Supplementary file 1 — Appendix S1: Deviations from the study protocol [file 12887_2025_5632_MOESM1_ESM.docx]

**Appendix S1. Deviations from the study protocol**

| **Protocol method** | **Deviations from protocol method, with justification** |
| --- | --- |
| We planned to include studies reporting a range of health outcomes (HbA1c, Time in range, Fasting blood glucose and Quality of life. | Prior to publishing our review another systematic review was published (16)with similar focus to our intended review, however they did not include QoL as an outcome. We believe QoL is an important aspect to consider in any intervention for T1 Diabetes. We therefore adjusted the focus of our review to QoL assessment.  This is still aligned to our initial aim. |
