# Supplementary figures and images for: Quality of life and physical activity in type 1 diabetes
Source: BMC Pediatr. 2025 May 1;25:345. doi: 10.1186/s12887-025-05632-6 (PMC12044773; doi:10.1186/s12887-025-05632-6)

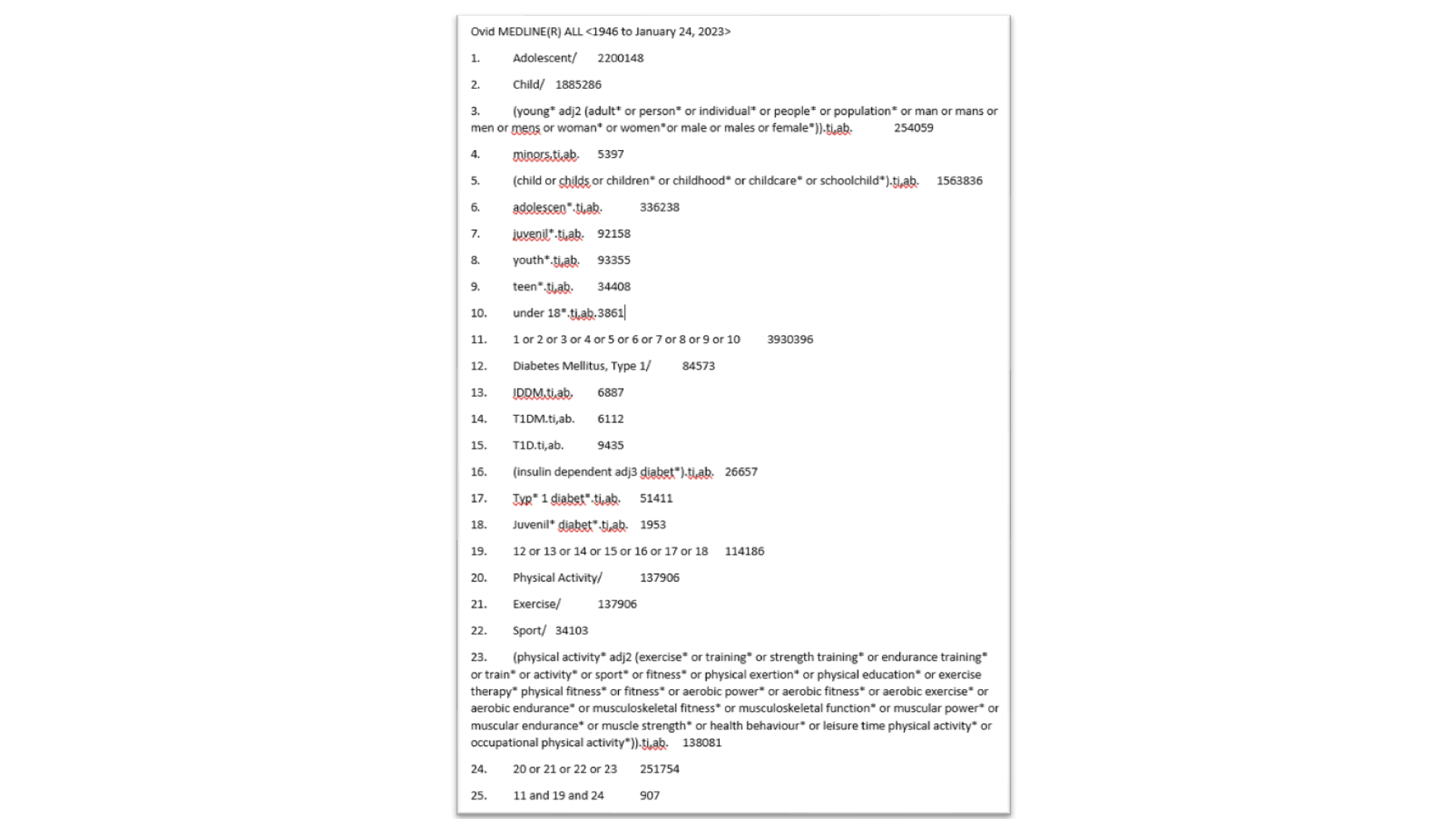

Supplement: Supplementary file 2 — Supplementary Figure 1: Search Strategy [file 12887_2025_5632_MOESM2_ESM.jpeg]
